# Pelvic Pain of Myofascial Origin in Women: Correlation with Lower Urinary Tract Symptoms

**DOI:** 10.1155/2024/5568010

**Published:** 2024-03-15

**Authors:** Sabrina Einig, Esther Ruess, Andreas Schoetzau, Kerstin Ayllon Bartet, Viola Heinzelmann-Schwarz, Francesco Vigo, Tilemachos Kavvadias

**Affiliations:** ^1^University Hospital of Basel, Basel, Switzerland; ^2^University of Basel, Basel, Switzerland

## Abstract

**Introduction:**

Women with lower urinary tract symptoms (LUTS) and high-tone pelvic floor often experience pain and have positive trigger points upon pelvic floor examination. However, the correlation of these findings has not yet been systematically examined and sufficiently understood. The aim of this cross-sectional study is to examine the correlation of pelvic myofascial pain with LUTS and pelvic floor tone.

**Materials and Methods:**

All participants filled a standardized pelvic floor questionnaire to assess LUTS, which consists of a total of 43 questions regarding bladder, bowel, and sexual function as well as prolapse symptoms. Myofascial trigger points in different muscle groups including pubococcygeus, iliococcygeus, and obturator as well as pelvic floor muscle tone were assessed using a standardized digital examination technique.

**Results:**

110 women were included in the study. There was a significant correlation between pain in various muscle groups and LUTS as well as high-tone pelvic floor muscle. A significant correlation could also be found between high pelvic floor muscle tone and the overall questionnaire score (*p* < 0.001) as well as the bladder function score (*p* < 0.001) and various pain scores of the different groups. Individuals with high-tone pelvic floor were more likely to have more LUTS and higher pain scores.

**Conclusions:**

The existence of myofascial pelvic floor trigger points and high pelvic floor muscle tone seem to be reflective of pelvic floor symptoms, as assessed with a standardized pelvic floor questionnaire.

## 1. Introduction

Myofascial pain syndrome is a syndrome of pain located in soft tissues, such as muscles and fascia. The pain can be local or referred and is provoked by (myofascial) trigger points. Local, regional, and generalized forms of soft tissue pain syndromes are described, whereas pain can occur independently or originate from other pain generators, such as trauma or inflammation [1]. Myofascial pain syndrome is characterized by the presence of myofascial trigger points (MTrPs), which are tender spots in the affected soft tissue and can cause pain upon palpation [2]. An MTrP consists of numerous contraction knots, which appear to be segments of muscle fibers with shortened and contracted sarcomeres. These areas of intense focal sarcomere contraction are palpable and are painful on compression [3].

MTrPs can be found in the pelvis and can affect various muscle groups, such as the levator ani muscle, the obturator internus, and the piriformis muscle [4]. There is evidence that women with pelvic floor symptoms and chronic pelvic pain (CPP) often experience myofascial pain and have positive trigger points upon pelvic floor examination [5]. CPP affects a significant number of women, with a prevalence, which is described to be between 5.7% and 26.6% worldwide and, although the etiology of CPP is multifactorial and not fully understood, it seems to be connected to myofascial pain with various possible pain generators, such as painful bladder syndrome and pelvic floor dysfunction [6]. Also, myofascial pelvic pain has been observed in women with lower urinary tract symptoms (LUTS) and other pelvic floor disorders such as pelvic organ prolapse (POP) [7].

Despite the high prevalence of CPP and its possible correlation with myofascial trigger points and LUTS, these conditions still remain often underevaluated and, accordingly, undertreated. In the current literature, physical examination methods used to evaluate pelvic floor muscle tone and myofascial pain vary significantly, and often, examination methods are undefined [8]. Although there is strong evidence that correct diagnosis and treatment of MTrPs is important for the treatment of pelvic floor symptoms, there is still a lack of studies that explore this correlation [9].

The aim of this study is to examine the correlation between myofascial trigger points and pelvic floor symptoms using a standardized pelvic floor examination method and a validated pelvic floor questionnaire.

## 2. Materials and Methods

### 2.1. Study Population

The study was performed in the outpatient urogynecological department of our clinic, and study participants were recruited during routine appointments. Some patients were referred to our center by physicians in private practice, and others presented themselves independently to our clinic or were referred by our in-house gynecologic team. Patients suffered from lower urinary tract symptoms such as incontinence, POP, or CPP.

We included adult female patients from the age of 18 years, regardless of parity, history of former urogynecologic treatment, or surgery. All participants gave written informed consent, and the project has been approved by the local regulatory authorities (ID 956).

### 2.2. Examination Method

Physical examination was performed by trained physicians using a standardized method. Study participants were examined in the lithotomy position. Digital examination was performed according to the proposal of the Pelvic Floor Clinical Assessment Group of the International Continence Society [10]. The assessment included the following:

- Evaluation of the pelvic floor muscle tone at rest
- Bilateral vaginal palpation of the anterior component of the levator ani muscle (M. pubococcygeus)
- Bilateral vaginal palpation of the posterior component of the levator ani muscle (M. iliococcygeus)
- Bilateral vaginal palpation of the obturator internus muscle
- Bimanual palpation of the empty bladder and urethra
- Evaluation of levator ani muscle contraction using the Oxford grading system on a scale from 0 (no contraction) to 5 (maximum contraction)

Pain was assessed using the visual analogue scale (VAS) upon palpation of the targeted muscle components. A VAS score of 0 equaled no pain; maximum pain was described by a VAS score of 10 [11].

Participants also filled out the standardized German version of the Australian pelvic floor questionnaire, which is a tool to evaluate severity and bothersomeness of pelvic floor symptoms [12]. It consists of a total of 43 questions regarding bladder, bowel, and sexual function as well as prolapse symptoms. The questionnaire provides a scoring system for each category (0–10) as well as a total score (0–40), whereas a higher score indicates greater symptoms.

Demographic data such as age, parity, and medical history including comorbidities and history of previous pelvic floor surgery were retrieved from the patients' medical records. The age-adjusted Charlson Comorbidity Index (CCI) was used to further define comorbidities of our study population [13].

### 2.3. Statistical Analysis

Descriptive statistics are presented as counts and frequencies for categorical data and median [Min, Max] for metric variables. Overall *p* values correspond to Kruskal–Wallis tests and chi-squared or exact Fisher test when the expected frequencies are less than 5 in some cells. Associations between ordinal variables are presented as spearman rank correlations. Values range between −1 and 1, where 0 means no correlation and values towards 1 or -1 means strong correlations. Positive values indicate positive associations, and negative values indicate negative associations. Associations between ordinal and categorical variables are presented as *p* values of Kruskal–Wallis tests except for high PMFT. In case of PMFT, polychoric correlation was used. A *p* value <0.05 is considered as significant.

All evaluations were performed using the statistical software R (Version 4.1.2).

## 3. Results

A total of 110 women were included in the study. The mean age was 55.9 (SD ± 17) years. Pelvic floor muscle tone was assessed as normal in 71 (64.5%) and high in 39 (35.5%) of the participants. The mean score of the pelvic floor questionnaire was 8.23 (SD ± 3.94). Pain upon palpation of the obturator, pubococcygeus, and iliococcygeus—but not of the bladder—was significantly correlated with the overall score of the pelvic floor questionnaire. High PFMT tone was significantly correlated with all the questionnaire domain scores (Table 1). Bladder pain upon palpation was correlated with age (*p*=0.001), the Charlson comorbidity index (CCI) (*p*=0.001), and a high PFMT. Obturator pain was correlated with parity (right: *p* < 0.001, left: *p*=0.035) but not with age, CCI, maximum birth weight, previous hysterectomy, or pelvic floor surgery. Also, pubococcygeus and obturatorius pain was significantly correlated with high pelvic floor muscle tone and parity (Table 2). High pelvic floor muscle tone was more frequent in parous than in nonparous women (85% vs. 62%, *p*=0.007) and in women who have had a hysterectomy, than in those with the intact uterus (43.5% vs. 21%, *p*=0.009) (Table 3). The mean overall pelvic floor questionnaire score (*p* < 0.001), as well as all individual domain scores, was significantly higher in women with high-tone pelvic floor (adjusted for CCI, parity, and previous hysterectomy) (Table 3).

## 4. Discussion

Our study highlights the correlation of pelvic myofascial pain with high pelvic floor muscle tone and lower urinary tract symptoms, using a standardized examination method and a validated pelvic floor symptom questionnaire. It supports and confirms a hypothesis that has been postulated in the literature before.

In our previous work from 2013, we showed that myofascial pelvic pain upon palpation in asymptomatic women should be considered as an uncommon finding [14]. It still remains unclear, under which circumstances women develop increased muscle sensitivity and myofascial pain in the pelvic region, but there is evidence that this hyperalgic condition is associated with various comorbidities, such as gynecological (endometriosis, dyspareunia, and vulvodynia), urological (painful bladder syndrome), gastrointestinal (irritable bowel syndrome and chronic constipation), and musculoskeletal (lumbar pain, joint dysfunction, osteoarthritis, and fibromyalgia) disorders and psychosocial stress factors (depression, anxiety, and sexual assault) [15, 16]. One possible reason is that the female pelvic floor is exposed to various mechanical stressors and hormonal changes, such as pregnancy, childbirth, surgery, and menopause, which manifest clinically at some point of a woman's life as pelvic floor dysfunction and lower urinary tract symptoms [17, 18]. It seems, also, that there is a link between these conditions and inflammatory reactions in the pelvic region (e.g., infections, endometriosis but also pathological conditions in adjacent organs such as the bowel and bladder) with the pelvic floor hypersensitivity and myofascial trigger points that can be found in these women [19]. Neural interactions, activation of certain central nervous system paths, and “crosstalk” between somatic and visceral afferent information may be the key to understanding the development of the symptoms. The co-occurrence of pain syndromes such as visceral pain, fibromyalgia, and musculoskeletal pain may imply a mutual mechanism of pain; however, the interplay between peripheral and central pain sensitivity, and especially of its evolution during lifetime, is still incomplete, and further future studies in both humans and experimental models are needed [20, 21].

**Figure 1:**
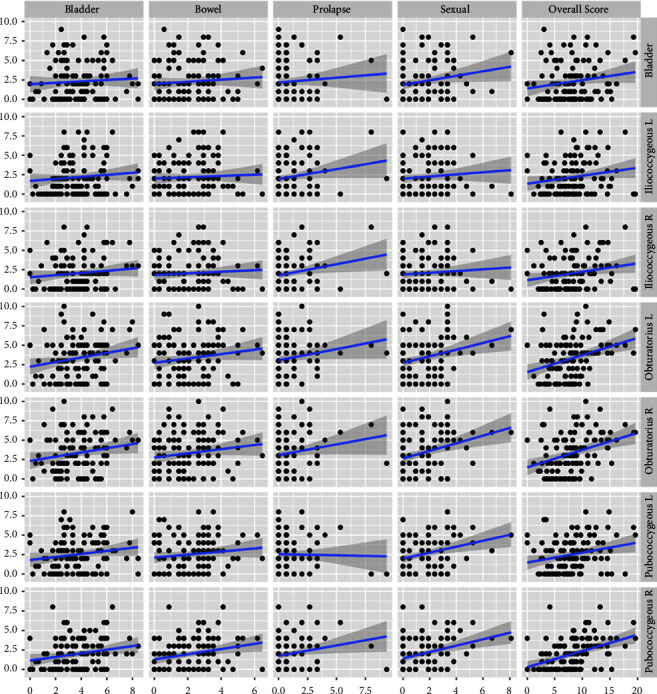
Correlations between pelvic myofascial trigger points (*y* axis) and pelvic floor questionnaire scores (*x* axis).

In our cohort, there was a significant correlation between myofascial pain in different regions of the pelvic floor and lower urinary tract symptoms. Although there were small differences in the correlation strength between different muscle groups and questionnaire domains, the trend was obvious in all our measurements (Figure 1). Interestingly, the obturator internus muscle was found to be excessively painful, mostly so in women, who have given birth (Table 1). There are already published reports of obturator internus muscle pain during pregnancy, which is associated with pelvic girdle pain, and also in women with chronic pelvic pain, although the exact pathophysiology of these findings is unknown [22, 23]. A possible explanation could be the involvement of the obturator internus muscle in the stability of the pelvis. Ackerman et al. report on a large cohort of women with frequency and urgency symptoms who also presented tenderness and pelvic floor hypertonicity, although the exact examination method was not described [24]. The authors suggest a novel phenotype of urinary symptoms named myofascial urinary frequency syndrome (MUFS), and they used electromyography to make the distinction between the “classical” overactive bladder/painful bladder syndrome—patients and those with myofascial dysfunction. Our finding underlines the importance of a thorough examination of the pelvic floor including muscle groups that are not routinely included in the palpation of the pelvis [25].

Another interesting finding of our analysis was the association of myofascial pain with high pelvic floor muscle tone. The latter is an issue that seems often underestimated or misunderstood and thus either ignored or misused. There is evidence that increased tone upon palpation of the pelvic floor is a finding associated with pelvic floor dysfunction, such as pain, sexual dysfunction, and incontinence [26, 27]. Although the examination (intrarater and interrater) reliability of the pelvic floor tone seems to be excellent (kappa values 0.95–0.98) [28], various terminologies, methodology, and design issues make the existing literature impossible to extract convincing arguments on the importance of this finding [29]. Volpe et al. in a prospective trial (RELAX trial) showed that 6 sessions of physical therapy in women with high-tone pelvic floor dysfunction not only increase the mean levator hiatal area (13.71 ± 1.77 cm^2^ vs 14.43 ± 2.17 cm^2^, *p*=0.05), suggesting relaxation and lengthening of the pelvic floor muscle, but also improve genitourinary symptoms, pain, lower gastrointestinal symptoms, and quality-of-life measures [30]. Meister et al. also report on myofascial pelvic floor pain and LUTS and found an association between bothersome symptoms and pain; however, they did not assess the pelvic floor tone, which seems to be of importance when assessing the pelvic floor function. [31]. Torosis et al., in a consensus report on treatment algorithms for patients with high-tone pelvic floor, highlight the need to address pelvic floor hypertonicity which should be included in the treatment algorithm [32]. The European Association of Urology in 2022 published an excellent paper on the importance of the correct and thorough evaluation, diagnosis, and clinical management of pelvic myofascial pain [9]. The authors highlight the importance of physical therapy and multidisciplinary approach in patients with this condition but also the need for further research on the topic, due to the lack of well-designed, quality studies.

The significance of our findings lies in the importance of a detailed evaluation of pelvic floor symptoms as well as the clinical evaluation of myofascial trigger points. In this case, the patient would benefit from a better understanding of their symptoms as well as from the opportunity of a holistic treatment, including not only her urinary symptoms but also the complex vicious cycle of pain. Based on our findings and our clinical experience, every woman with lower urinary tract symptoms should be offered a screening for myofascial pelvic pain and examination of the pelvic floor muscle tone from an experienced clinician or physiotherapist. However, there are limitations to our study. First, there is a lack of a power analysis, and the number of included patients was relatively small. Second, we do not have data on possible factors that could bias the results and pain sensitivity, such as medication intake, history of endometriosis in the premenopausal patients, or history of chronic pain conditions, such as fibromyalgia or chronic lumbar pain. However, our paper presents the typical unfiltered patient cohort with pelvic floor dysfunction, who seeks medical help in a tertiary hospital setting and thus corresponds well to real-life conditions.

## 5. Conclusion

Myofascial pelvic floor trigger points are reflective of pelvic floor symptoms, as assessed with a standardized pelvic floor questionnaire. Examination in women with lower urinary symptoms should include diagnostics for pelvic floor muscle pain in order to optimize clinical diagnosis and offer appropriate treatment.

## Figures and Tables

**Table 1:** Correlation between maofascial pain scores and questionnaire domain scores.

	Questionnaire domain scores				
	Bladder	Bowel	Prolapse	Sexual	Overall score
Myofascial pain location					
(i) Pubococcygeus R	**0.22 (0.018)**	**0.26 (0.004)**	**0.31 (<0.001)**	**0.38 (<0.001)**	**0.44 (<0.001)**
(ii) Pubococcygeus L	0.15 (0.10)	0.12 (0.18)	0.05 (0.53)	**0.31 (<0.001)**	**0.24 (0.01)**
(iii) Iliococcygeus R	0.13 (0.15)	0.06 (0.49)	**0.24 (0.009**)	0.11 (0.21)	**0.21 (0.02)**
(iv) Iliococcygeus L	0.14 (0.13)	0.04 (0.63)	**0.20 (0.03)**	0.14 (0.13)	**0.19 (0.04)**
(v) Obturatorius R	**0.17 (0.06**)	0.15 (0.11)	**0.20 (0.03)**	**0.34 (<0.001)**	**0.32 (<0.001)**
(vi) Obturatorius L	**0.19 (0.04)**	0.16 (0.08)	**0.24 (0.01)**	**0.29 (0.002)**	**0.32 (<0.001)**
(vii) Bladder	0.51 (0.32)	0.44 (0.22)	0.35 (0.18)	**0.43 (0.02)**	**0.69 (0.05)**
High-tone pelvic floor	**0.51 (<0.001)**	**0.44 (<0.001)**	**0.35 (<0.001)**	**0.43 (<0.001)**	**0.69 (<0.001)**

Correlations and *p* values (in brackets) between myofascial pain locations and questionnaire scores as well as between high tone pelvic floor and questionnaire scores (Spearman's rank correlation). In case of high-tone pelvic score, polychoric correlation was calculated. Significant correlations are presented in bold. R: right, L: left.

**Table 2:** Associations of myofascial pain scores and patients' characteristics.

	Patients' characteristics					
	Age	CCI	Parity	Max. BW	Pelvic floor surgery	High PFMT
Myofascial pain location						
(i) Pubococcygeus R	0.476 (0.06)	0.009 (0.009)	0.11 (0.30)	0.03 (0.839)	0.10 (0.124)	**0.34 (<0.001)**
(ii) Pubococcygeus L	0.06 (0.315)	0.05 (0.573)	0.10 (0.261)	0.27 (0.094)	0.12 (0.40)	**0.40 (<0.001)**
(iii) Iliococcygeus R	0.07 (0.412)	0.07 (0.441)	0.09 (0.046)	0.06 (0.698)	0.08 (0.303)	**0.25 (0.008)**
(iv) Iliococcygeus L	0.05 (0.573)	0.03 (0.734)	0.19 (0.339)	0.14 (0.370)	0.11 (0.639)	**0.22 (0.018)**
(v) Obturatorius R	**0.23 (0.014)**	0.17 (0.068)	**0.32 (0.008)**	0.06 (0.863)	0.02 (0.116)	**0.29 (0.002)**
(vi) Obturatorius L	0.10 (0.255)	0.10 (0.280)	**0.20 (0.035)**	0.02 (0.691)	0.10 (0.178)	**0.32 (<0.001)**
(vii) Bladder	**0.3 (0.001)**	**0.29 (0.001)**	0.04 (0.619)	0.08 (0.609)	0.18 (0.909)	**0.24 (0.01)**

Associations (*p* values) of myofascial pain scores and patients' characteristics (*N* = 110). For associations with age, CCI, and max. BW Spearman rank correlation coefficient was used. For associations with parity, the Kruskal–Wallis test was used. For high PMFT, polychoric correlation was used. The significant correlations (*p* < 0.05) are presented in bold. CCI: Charlson Comorbidity Index. BW: Birth weight. PFMT: pelvic floor muscle tone.

**Table 3:** Characteristics and mean questionnaire scores in women with high and normal pelvic floor muscle tone.

	High PFMT (*n* = 39)	Normal PFMT (*n* = 71)	*p* value
Age (mean, SD)	57.1 (±17.8)	53.6 (±15.3)	0.29
Hysterectomy, *n* (%)	17 (43.5%)	15 (21%)	**0.009**
Previous pelvic floor surgery	7 (17.8%)	9 (12.5%)	0.16
Parity, *n* (%)	33 (85%)	44 (62%)	**0.007**
(i) C. section (%)	8 (24%)	15 (21%)	0.16
(ii) Instrumental delivery (%)	6 (15%)	7 (10%)	0.09
(iii) Birth weight, gr (mean, SD)^*∗*^	3529 (±500)	3605 (±595)	0.67
Questionnaire score (mean, SD)	11.18 (±2.9)	6.61 (±3.7)	**<0.001** ^*∗∗*^
(i) Bladder score	4.68 (1.76)	3.18 (1.18)	**<0.001** ^*∗∗*^
(ii) Bowel score	2.81 (1.48)	1.79 (1.26)	**0.001** ^*∗∗*^
(iii) Prolapse score	2.10 (1.49)	0.63 (1.05)	**0.002** ^*∗∗*^
(iv) Sexual domain score	2.02 (2.20)	1.01 (1.23)	**0.001** ^*∗∗*^

Characteristics and mean questionnaire scores in women with high and normal pelvic floor muscle tone (PFMT) as assessed by digital examination. *p* values of characteristics reported in % are based on the chi-squared test (age, previous hysterectomy, previous pelvic floor surgery, and parity); *p* values for the questionnaire scores were reported based on the Kruskal–Wallis test. ^*∗*^Birth weight in case of vaginal delivery. ^*∗∗*^Adjustments for the following confounders were made: CCI, parity, and previous hysterectomy.

## Data Availability

Data are available upon request.
